# Impact of scribing history and physical notes and procedure reports on endoscopist efficiency during routine procedures: a proof-of-concept study

**DOI:** 10.1038/s41424-018-0042-3

**Published:** 2018-08-10

**Authors:** Margaret E. MacPhail, Samuel A. Main, William W. Tippins, Andrew W. Sullivan, Douglas K. Rex

**Affiliations:** Department of Medicine, Division of Gastroenterology/Hepatology, Indiana University School of Medicine, Indianapolis, IN USA

## Abstract

**Background:**

Efficiency is an important aspect of endoscopic practice that has received limited study. We evaluated the impact of scribing electronic pre-procedure history and physical examinations, and electronic procedure reports on endoscopist efficiency.

**Methods:**

We used a stopwatch to measure the time between the procedures (scope out to scope in), pre-procedure patient assessment time, and procedure report generation time for 180 consecutive procedures performed by a single endoscopist with or without a scribe for recording history and physical and procedure reports. Schedulers were unaware of whether a scribe would be present.

**Results:**

Mean times for recording the pre-procedure history and physical and procedure reports were reduced by 34% (*p* = 0.001) and 71% (*p* < 0.0001), respectively, when scribes were used. The mean time saved by the endoscopist from scribing the history and the physical and procedure reports was 2.12 and 1.59 min, respectively. When both processes were scribed, the endoscopist spent 42% (*p* = 0.033) longer in the recovery area (absolute mean increase 1.01 min) compared with when no scribes were utilized. The total time saved per 6.5-h procedure block with both scribes averaged to 41.7 min.

**Conclusion:**

The use of scribes to record history and physical examination notes and procedure reports saved enough endoscopist time to allow additional procedures or longer procedures, or to free the time for other tasks.

## Introduction

Endoscopic procedures are among the most commonly performed medical procedures^1–4^. Demand is particularly high for routine upper endoscopy and colonoscopy^1,2^. With increasing demand for endoscopic services as a result of an aging population and improved screening and surveillance practices^5,6^, there is a need to establish systems and practices that allow for high-quality endoscopy and efficient delivery of procedures.

In 2007, there were 3.9 active gastrointestinal physicians per 100,000 people in the United States^7^. Most other countries have fewer gastroenterologists per capita^7^. Available data suggest that endoscopy delivery is often inefficient. Yang et al.^8^ found that non-endoscopy time comprised 67.2% of total endoscopic time per day. In a previous study, we showed that room turnover time is a key determinant of efficiency^9^. To our knowledge, no studies have investigated the impact of scribes on endoscopist efficiency.

In some endoscopy units, physician activity between the procedures is the rate-limiting determinant of efficient performance. After an endoscopy is completed, usual physician activities include generating a procedure report, visiting the recovery area to see and discharge a previously endoscoped patient, and visiting an assessment area to perform a history and physical examination and obtaining informed consent for an upcoming procedure. In this prospective study, we sought to evaluate the impact on efficiency of using scribes to electronically record the history and physical examination in the assessment area and to generate the electronic procedure report. Because the study was performed in the practice of a single expert endoscopist, we consider this as a proof-of-concept study.

## Materials and methods

All endoscopic procedures were performed by D.K.R. at a surgery center in Carmel, Indiana. Endoscopic sessions were conducted beginning at 6 am and lasting until 12:30 pm. All scheduled procedures were colonoscopies or upper endoscopies, with occasional pouchoscopies or flexible sigmoidoscopies. There were no endoscopic ultrasound or endoscopic retrograde pancreatography procedures. Upper endoscopies were routinely scheduled for 15 min, with occasional 30-min procedures for resection of duodenal adenomas. Colonoscopies were scheduled at 30-min intervals, with occasional procedures scheduled for 45 min for resectioning a large polyp identified by a referring physician, or performing colonoscopy in a patient with a previous attempt of colonoscopy by another physician who did not complete cecal intubation.

The endoscopist reviewed all scheduled procedures the evening before and identified the procedure indications, the results of prior procedures, and major medical issues using the electronic medical records. The endoscopy unit is open access, and consents were obtained only on the day of the procedure. The endoscopist was assigned two procedure rooms each day. Prior to the first procedure at 6 am, D.K.R. evaluated two patients in the assessment area and recorded their history and physical examinations electronically (Cerner; North Kansas City, MO). When no scribe was present, D.K.R. entered the assessment room, introduced himself, and logged on to Cerner. The history and physical examination were performed and recorded on Cerner concurrently and finalized by D.K.R. before leaving the assessment room. Informed consent was verbally obtained and signed on paper by D.K.R. and the patient. After the first procedure was completed, the endoscopy report was generated using Provation (Provation Medical; Minneapolis, MN). The study endoscopist then returned to the assessment area to perform the history and physical examination and to obtain consent from the third patient on the schedule. Thus, the history and physical examinations and the consents were always completed for the two patients who were just ahead on the schedule of the patient actively undergoing a procedure. This was necessary because the unit policy was that the history and physical electronic note and the informed consent be finalized before a patient could be brought to the procedure room. Once the history and physical examination and the consent were obtained in the assessment area from the third patient, D.K.R. returned to the second procedure room and performed the second endoscopic procedure. After the second procedure was completed, he went to the recovery area to evaluate and authorize the discharge of the patient who underwent the first procedure. After this, the cycle of activity continued as the assessment area, the endoscopic procedure room, then the recovery area, and so on. No gastroenterology fellow was involved in any aspect of any pre-procedure assessment, endoscopic procedure, or any other aspect of patient care on the day of the procedure.

The use of two endoscopic procedure rooms meant that the room turnover in one procedure room was almost always completed, and the next patient was ready for endoscopy before D.K.R. completed the procedure in the other room and cycled to recovery and assessment and back to the other procedure room. Nearly all patients underwent sedation and all sedations were administered by an anesthesiologist (no certified registered nurse (RN) anesthetists are employed at the center). One anesthesiologist was assigned to work with D.K.R. each day. The anesthesiologist met each patient in the procedure room (rather than in the assessment area) and performed their own history and physical examnination and obtained consent for sedation in the procedure room. The anesthesiologist cycled only between the procedure rooms and the recovery area. All the anesthesiologists had previously demonstrated sufficient efficiency to stay ahead of D.K.R. in the room cycling process.

When scribes were present, D.K.R. verbally gave the scribe all pertinent findings during the procedure. Immediately following completion of the procedure, D.K.R. removed the mask, gown, and gloves, handwashed, and proceeded to the recovery area or assessment, depending on the point in the schedule. Before returning to the next procedure room, D.K.R. reviewed the previous Provation report created by scribe for accuracy and signed it. When a scribe was present for the history and physical examination in the assessment area, D.K.R. entered the assessment room, reviewed the key history, and performed a physical examination and obtained the informed consent, but did not log on to Cerner or electronically record the history and physical examination.

All scheduling was done by endoscopy unit employees without the knowledge of whether a scribe would be available. Scribes were employees of Indiana University and were members of research team of D.K.R. They were trained to scribe prior to study onset by D.K.R. He performed the procedures in the Carmel endoscopy unit 2 days per week. In general, we alternated the order of the days each week, in which scribes were used versus not used. There were some days when a scribe was not available because of vacation, etc. The scribe for procedure recording started work at 6 am and was available on their assigned day, beginning with the first procedure The procedure scribe had a Bachelor of Arts degree. The scribe who was recording the history and physical examinations started work at 7:30 am and thus was not available for the first three to four procedures on the day she was assigned. One individual, a licensed RN, performed all the history and examination scribing.

Time was measured by a research assistant using a stopwatch. Only the time spent by D.K.R. in various tasks was recorded. The history and physical time was from when D.K.R. left the recovery area until he left a patient’s assessment room. Procedure report time was from D.K.R. initially touching the computer to signing the report. Recovery room time was from when D.K.R. left the procedure room until he left the recovery area. Total time between the procedures was from the scope out of the last procedure until the scope in of the next procedure.

The data were collected as part of assessment to determine the value of hiring permanent scribes for the endoscopy unit. Permission to review the de-identified data for publication was granted with exempt status by the IRB on 6 December 2017.

After completion of the study, 10 history and physical notes done by the scribe and 10 history and physical reports done by D.K.R. were randomly selected and then blindly evaluated by D.K.R. for quality assurance. The number of abnormal history and examination findings in each report were counted and used as a quality measure. Procedure reports were analyzed for quality by randomly selecting 10 reports done by D.K.R. and 10 reports created initially by the scribe. The reports were blindly evaluated by D.K.R. for quality based on whether the language was appropriate, typing errors were present, and recommendations were appropriate to the findings.

Time end points were compared between the groups (with and without scribes), using analyses of variance and two-sample *t*-tests.

## Results

We measured the study times on 16 days, with an average of 11.25 patients per day. The history and physical note scribe was present for 13 days of data collection for a fraction of the 6.5-h period. The procedure report scribe was present for the entire 6.5-h endoscopy period for 8 days throughout data collection. Of 180 procedures studied, 134 were colonoscopies, 29 were esophagogastroduodenoscopy (EGDs), and the remaining procedures included flexible sigmoidoscopy, pouchoscopy, and combined upper and lower endoscopy.

The endoscopist completed 88 history and physical notes, while the scribe completed 92 notes. When a scribe was present to complete the history and physical notes, the mean assessment time decreased by 34% (*p* = 0.0001) or a mean of 1.59 min per patient (Table 1). This difference was consistent across different procedure types.

**Table 1:** Percent difference between the mean time endoscopist spent on each phase in the presence of scribes and in the absence of scribes

Interval measured	Mean time without scribes (min)	Mean time with scribes (min)	Difference in mean times (min):	% Change in time	*p*-value
Procedure time	19.55	20.99	1.44	7	0.474
Assessment time	4.66	3.07	−1.59	−34	0.001
Recovery room time	2.39	3.40	1.01	42	0.033
Procedure report time (all)	3.00	0.88	−2.12	−71	<0.001
Colonoscopy procedure report time	2.91	0.63	−2.28	−78	<0.001
EGD procedure report time	3.26	1.84	−1.43	−44	0.011
Total time between procedures	12.61	13.54	0.92	7	0.257

The procedure report scribe completed 94 procedure reports, while the endoscopist completed 88. Table 1 shows that endoscopist time spent on procedure report decreased by 71% with scribing of procedure reports (*p* < 0.0001). Mean endoscopist time on colonoscopy reports was reduced by 78% on average or a mean of 2.38 ± 0.84 min per patient (*p* < 0.0001). Mean time spent on EGD procedure reports was reduced by 44% or a mean of 1.43 ± 2.05 min per procedure (*p* = 0.011).

With scribing, mean recovery room time increased from 2.39 to 3.40 min or by 42% (*p* = 0.03). When only the procedure scribe was present, there was still a significant (*p* = 0.008) increase in mean recovery room time, from 2.16 to 3.06 min.

Mean procedure time for colonoscopies was 22.59 ± 7.80 min, range 8.73–59.40 min, while the EGD procedures had a mean time of 10.28 ± 7.51 min, range 2.78–41.27 min. There was a non-significant increase in mean procedure time from 19.55 to 20.99 with the use of scribes (*p* = 0.383). This added ~7%, or 1.44 min, to each procedure (Table 1).

The change in mean times between procedures (scope out to scope in) when a scribe was writing procedure reports (13.54 min) versus when the endoscopist was writing reports (12.61 min) was not statistically significant (*p* = 0.257). The mean time between procedures was not different when the previous procedure was an EGD (12.69 min) versus when the previous procedure was a colonoscopy (12.35 min).

No patient suffered a complication during any of the study procedures.

Quality assessment of history and physical notes identified that a mean of 4.0 abnormal findings was reported per patient when a scribe completed the electronic recording, compared with a mean of 3.5 abnormal findings per patient when the physician wrote the history and physical note. Procedure reports created by scribes versus endoscopist were not different when evaluated for quality.

## Discussion

Greater endoscopic efficiency could help meet the rising demand for endoscopic procedures. We found that utilizing scribes to complete the pre-procedure history and physical notes and procedure reports significantly decreased the time to complete these tasks, compared with the endoscopist recording this information. The absolute reduction in time spent by the endoscopist scribing procedure reports was 2.12 min per case and the mean reduction in time spent by the endoscopist in recording the history and physical notes was 1.59 min, or a total mean time savings for the endoscopist was 3.71 min per procedure. With an average of 11.25 cases performed per 6.5-h session, this time saved would allow performing an additional colonoscopy per session. We noted an increase in recovery room time per case when scribes were used and a non-significant trend toward longer procedure times. Thus, the use of scribes might result in greater patient satisfaction (e.g., more time spent in the recovery room answering questions) and higher quality procedures. It’s likely that the study endoscopist felt less pressure to move through the procedures and the recovery rooms visits in the presence of scribes and subconsciously spent longer on these aspects of patient care. It might require actually shortening the amount of time allotted on the schedule per procedure to force an endoscopist to achieve the procedure times and recovery times achieved when the scribes are not present, and thus achieve the economic advantages of a shorter work session or an additional procedure per session.

We saw a greater savings in time for the endoscopist with procedure report scribing of colonoscopy reports compared with EGD reports. Our anecdotal impression is that colonoscopy reports are more “cook-book” with standardized recording of most findings in patients without inflammatory bowel disease. In screening, surveillance, and diagnostic colonoscopies, most findings can be selected from the drop-down menus in Provation. Upper endoscopy reports often require more typing by the endoscopist to portray the findings accurately and convey recommendations to the referring physician.

Our study included several limitations. First, we only measured time intervals for one physician. Multiple physicians would provide an analysis that could be more widely applicable. However, the study endoscopist is generally focused on efficiency and attempts to run a high-volume, high-quality endoscopic practice. Interprocedure times in this study were at the lower end of the observed turnover times, (interprocedure times and turnover times are not the same, since turnover time is usually applied to the turnover in a single room)^9^, which is consistent with this suggestion. Thus, the study endoscopist's experience with scribes likely reflects what can be expected from the use of scribes in an efficiency-focused practice. Despite this, we acknowledge that our study should be viewed as a proof-of-concept study, with a need for testing by other endoscopists in other settings. Second, the study endoscopist’s practice is set up so that the rate-limiting step in endoscopy efficiency is the endoscopist. Thus, two procedure rooms are used by the endoscopist, so waiting for room turnover in an individual room is rarely necessary. Also, the anesthesiologists are consistently able to rotate from one procedure room to recovery and back to the other procedure room before the endoscopist can cycle from one procedure room to the other. For units that do not have these features, the use of scribes might not create efficiencies for the endoscopy unit. However, in this regard, the study endoscopist is not the only endoscopist whom we consider sufficiently efficient and with high enough procedure volumes to warrant the use of two procedures rooms. Further, the senior endoscopist (D.K.R.) is anecdotally aware of endoscopists in other centers using the two-room model. Thus, we know that at least some other physicians are utilizing the two-room model and might benefit from this approach. Further, in some circumstances, use of scribes could improve the physician's efficiency when one room is utilized. Next, Provation software requires exiting “image capture” mode to enter procedure findings in the report. Therefore, the endoscopist is unable to take pictures if a scribe exits the image capture mode to record findings in the report as they are identified. Thus, the procedure proceeds most efficiently when the program remains in the image capture mode for the duration of the procedure. If the scribe could create the report while the program remained in the image capture mode, some additional efficiency might be achieved. However, real-time voice-activated procedure reports that could be completed by the endoscopist during the procedure could obviate any need for a procedure report scribe. Finally, we have not conducted a cost analysis of using scribes. At first glance, it seems questionable whether hiring two scribes would be cost-effective. However, in the current study, the scribes were research assistants of the senior author and were engaged in other productive work when not scribing. Our current two-room system is fashioned to make the endoscopist the rate-limiting step in efficiency. This means the RN in each room is generally waiting for the endoscopist to complete the procedure in the other room, which also means this RN could be trained to scribe the procedure report. Similarly, nurses in the assessment area could be trained to scribe the history and physical examination. In the latter two models, no extra costs for the practice would be incurred. Finally, we had D.K.R. blindly review the procedure reports, histories, and physical exminations for quality. This step might have been better delegated to another physician. However, all reports are electronic and in general have no identification features that would unblind the review.

We acknowledge that maximizing efficiency (versus reasonable efficiency or no emphasis on efficiency) is unlikely to be of interest to all endoscopists. In some settings, e.g., employed and salaried endoscopists, endoscopic efficiency may be of greater interest to healthcare administrators than to endoscopists. Maximizing endoscopic efficiency might increase physical stress or fatigue in endoscopists or in some cases result in anxiety or emotional stress. Increased efficiency would seem to be of greatest interest to physicians with high workloads who are paid on a productivity basis, such as per relative value update unit completed. Additional study is needed to understand who benefits most from efforts to maximize efficiency, as well as the risks of maximizing efficiency. As already noted, we consider it a fundamental tenet that striving for efficiency never results in rushing procedures or sacrificing the quality of endoscopic procedures.

In summary, our results suggest that scribes for electronic history and physical recording and procedure report recording can provide efficiencies that could increase procedure volume or allow time to lengthen procedures or improve other aspects of patient care. Our data provide a sense of the time savings an endoscopist might realize from use of scribes to electronically record history and physical examinations and procedure reports.

## Study highlights

### WHAT IS CURRENT KNOWLEDGE

- Efficient endoscopy has value to practitioners and society.
- The impact of scribing history and physical examinations and procedure reports on endoscopy efficiency is unknown.

### WHAT IS NEW HERE

- Scribes reduced the time to record history and physical examinations by 34% and procedure reports by 71%, compared to completion by a colonoscopist.
- Over a 6.5 h session, note scribing saved the endoscopist an average of 41 min—enough time to schedule an additional procedure or complete other tasks.
